# Mild White Matter Changes in Un-medicated Obsessive-Compulsive Disorder Patients and Their Unaffected Siblings

**DOI:** 10.3389/fnins.2015.00495

**Published:** 2016-01-11

**Authors:** Siyan Fan, Odile A. van den Heuvel, Danielle C. Cath, Ysbrand D. van der Werf, Stella J. de Wit, Froukje E. de Vries, Dick J. Veltman, Petra J. W. Pouwels

**Affiliations:** ^1^Department of Anatomy and Neurosciences, VU University Medical CenterAmsterdam, Netherlands; ^2^Department of Psychiatry, VU University Medical CenterAmsterdam, Netherlands; ^3^Department of Social and Behavioural Science, Utrecht UniversityUtrecht, Netherlands; ^4^Neuroscience Campus Amsterdam, VU/VU University Medical CenterAmsterdam, Netherlands; ^5^The OCD Team, Haukeland University HospitalBergen, Norway; ^6^Academic Anxiety Center AltrechtUtrecht, Netherlands; ^7^Netherlands Institute for NeuroscienceAmsterdam, Netherlands; ^8^Department of Physics and Medical Technology, VU University Medical CenterAmsterdam, Netherlands

**Keywords:** diffusion tensor imaging, fractional anisotropy, obsessive-compulsive disorder, endophenotype

## Abstract

**Objective:** Obsessive-compulsive disorder (OCD) is a common neuropsychiatric disorder with moderate genetic influences and white matter abnormalities in frontal-striatal and limbic regions. Inconsistencies in reported white matter results from diffusion tensor imaging (DTI) studies can be explained, at least partly, by medication use and between-group differences in disease profile and stage. We used a family design aiming to establish whether white matter abnormalities, if present in un-medicated OCD patients, also exist in their unaffected siblings.

**Method:** Forty-four OCD patients, un-medicated for at least the past 4 weeks, 15 of their unaffected siblings, and 37 healthy controls (HC) underwent DTI using a 3-Tesla MRI-scanner. Data analysis was done using tract-based spatial statistics (TBSS). Fractional anisotropy (FA), axial diffusivity (AD), radial diffusivity (RD), and mean diffusivity (MD) values were compared within seven skeletonised regions of interest (ROIs), i.e., corpus callosum, bilateral cingulum bundle, bilateral inferior longitudinal fasciculus/frontal-occipital fasciculus (ILF/FOF) and bilateral superior longitudinal fasciculus (SLF).

**Results:** Un-medicated OCD patients, compared with HC, had significantly lower FA in the left cingulum bundle. FA was trend-significantly lower in all other ROIs, except for the corpus callosum. Significant three-group differences in FA (and in RD at trend-significant level) were observed in the left cingulum bundle, with the unaffected siblings representing an intermediate group between OCD patients and HC.

**Conclusions:** OCD patients showed lower FA in the left cingulum bundle, partly driven by trend-significantly higher values in RD. Since the unaffected siblings were found to be an intermediate group between OCD patients and HC, this white matter alteration may be considered an endophenotype for OCD.

## Introduction

Obsessive-compulsive disorder (OCD) is a debilitating neuropsychiatric disorder characterized by obsessions (intrusive recurrent thoughts) and/or compulsions (repetitive behaviors). OCD is moderately heritable (Hudziak et al., 2004; Van Grootheest et al., 2007), with heritability rates between 40 and 45% (Hudziak et al., 2004) and first degree relatives of the patients having a 4–10 times higher risk of developing OCD (Nestadt et al., 2000), depending on age of the proband. At the same time, the genetic basis of OCD is complex, multi-factorial, and under strong environmental influence (Grisham et al., 2008). Both structural and functional neural correlates of OCD have been found in the unaffected first-degree relatives of OCD patients (Chamberlain et al., 2008; Menzies et al., 2008b; de Wit et al., 2012; de Vries et al., 2014), suggesting that at least in part, the alterations are state-independent and might be regarded as correlates of genetic vulnerability, also called endophenotypes (Gottesman and Gould, 2003).

In contrast to the large literature on gray matter alterations in OCD (Radua and Mataix-Cols, 2009; Rotge et al., 2009; de Wit et al., 2014), white matter abnormalities in OCD have caught relatively less attention. A recent OCD Brain Imaging Consortium mega-analysis (de Wit et al., 2014), involving 412 OCD patients and 368 healthy controls (HC), reported decreased white matter volumes in frontal regions in the patient group, suggesting abnormalities of white matter connections between the prefrontal and subcortical regions within the frontal-striatal circuits. These results were consistent with some previous studies on white matter volume (van den Heuvel et al., 2009; Togao et al., 2010). White matter volume alterations have also been reported for the parietal and occipital lobes (Riffkin et al., 2005; Lázaro et al., 2009, 2011).

Although potentially related to white matter volume, the microstructure of the white matter tracts may provide additional insights into the pathophysiology of OCD. Diffusion tensor imaging (DTI) is a widely used neuroimaging technique to study brain tissue microstructure by quantification of the diffusion characteristics of water molecules (Le Bihan et al., 2001). Anisotropy, generally expressed as fractional anisotropy (FA), is a directionally dependent property of water diffusivity. FA in white matter reflects the underlying characteristics of microstructure, such as fiber density, axonal diameter, thickness of the myelin sheaths, and directionality of the fibers (Koch et al., 2014). FA is derived from the three eigenvalues of the diffusion tensor: λ1, λ2, and λ3. The largest eigenvalue (λ1), or axial diffusivity (AD), has been found to be a possible marker for axonal injury. The average of the two smaller eigenvalues λ2 and λ3, or radial diffusivity (RD) has been suggested as an indicator of myelin damage (Song et al., 2003; Fan et al., 2012). Mean diffusivity (MD) is the average of all three eigenvalues.

A recent DTI meta-analysis by Radua et al., comparing 204 OCD patients with 231 matched HC, showed reduced FA in the corpus callosum, the cingulum bundle, the inferior longitudinal fasciculi (ILF)/the frontal-occipital fasciculi (FOF), and the superior longitudinal fasciculi (SLF). Whether these alterations reflect state or trait effects (i.e., are the result of adaptive changes as a consequence of disease) or underlie disease vulnerability (and thus can be considered an endophenotype of OCD), cannot be deduced from the classical case-control studies. Therefore, extension to a family-based approach is warranted.

Menzies et al. (2008b) were the first to show that both OCD patients and their first-degree relatives had reduced FA in the right inferior parietal white matter and increased FA in the right medial frontal region. However, their results may have been partly confounded by suboptimal matching between the groups and medication use. Also, they did not explore differences in diffusivity parameters underlying the FA alterations. A combined DTI/voxel-based morphometry study using a monozygotic (MZ) twin design reported white matter characteristics of MZ twins who were concordant and discordant on obsessive-compulsive traits (den Braber et al., 2011). This study reported effects for environmental as opposed to genetic influences on regional white matter volume: the predominant FA decrease found in inferior frontal regions in the MZ concordant-high vs. concordant-low twins was suggested to reflect genetic (trait) effects, whereas the within MZ discordant twin comparison revealed increased dorsolateral prefrontal white matter changes in the high scoring twins, thought to reflect changes due to environmental effects. More recently, Shaw et al. investigated adult and adolescent OCD patients and their unaffected siblings and found similar morphological abnormalities in cortical and subcortical regions of caudate nucleus, thalamus and the right orbitofrontal cortex. Besides, both OCD patients and unaffected siblings, as compared with healthy controls, showed increased thickness of the right precuneus (Shaw et al., 2015).

In summary, available evidence for white matter abnormalities in OCD is largely inconsistent. We suggest several possible reasons for the reported discrepancies: (1) in OCD, white matter alterations might be subtle thus difficult to detect; (2) medication effects seem to confound the findings (Yoo et al., 2007; Fan et al., 2012; Benedetti et al., 2013; Radua et al., 2014); (3) results are highly variable across pediatric, adolescent and adult patients due to changes in white matter architecture throughout brain development (Peters et al., 2012); (4) debate has risen recently on whether FA alone is sufficient and sufficiently representative to indicate changes in white matter microstructure (Hasan, 2006; Fan et al., 2012; Szczepankiewicz et al., 2014); and (5) small samples and inconsistent methodologies have been used (Radua et al., 2014).

In this study, we aimed to replicate and extend previous findings, by exploring white matter microstructure in a group of un-medicated adult OCD patients, their unaffected siblings and gender and age-matched HC. By using tract-based spatial statistics (TBSS), we first aimed to investigate whether there were any abnormalities of FA in OCD patients compared with HC, and to investigate whether these between-group differences could be explained by any of the diffusivity measures (i.e., AD and RD). Finally, we aimed to explore whether alterations in diffusion parameters also existed in the unaffected siblings, to disentangle whether white matter changes are cause or consequence of the disease.

We used a region-of-interest (ROI) approach by selecting 7 ROIs, based on the results from the meta-analysis of Radua et al. (2014), corpus callosum, the bilateral cingulum bundle, the bilateral ILF/FOF and the bilateral SLF. We hypothesized that OCD patients compared with HC would show lower FA with changes in diffusivity values in the ROIs. Moreover, based on the familiality of OCD we expected that these abnormalities would be shared (as a trait), at least partly, in the unaffected siblings of the patients.

## Methods and materials

### Participants

Forty-four OCD patients who were un-medicated for at least 4 weeks when participating in the study (mean age 38.5 year, SD = 9.9), 15 of their unaffected siblings (mean age 38.1 year, SD = 14.1), and 37 HC (mean age 39.5 year, SD = 11.5) were included. The groups were matched on age, gender, handedness, and education level. OCD patients were recruited from the outpatient clinics within the Netherlands OCD Association cohort (Schuurmans et al., 2012), the Academic Anxiety Center Altrecht (Utrecht, the Netherlands), and by online advertisements. HC were recruited by local and online community advertisements.

All participants were screened on axis I psychiatric disorders using the Structured Clinical Interview for DSM-IV-TR Axis I Disorders (First et al., 2002). OCD symptom characteristics and severity were assessed with the Yale-Brown Obsessive Compulsive Scale (Y-BOCS, symptom list and severity scale) (Goodman et al., 1989) and the/Inventory-Revised (OCI-R) (Foa et al., 2002). Depressive symptoms were assessed with the Montgomery-Åsberg Depression Rating Scale (MADRS) (Montgomery and Asberg, 1979) and handedness with the Edinburgh Handedness Inventory (Oldfield, 1971).

Current psychoactive medication use, current or past psychosis, major physical illness, a history of a major neurological illness, and MRI contra-indications served as exclusion criteria. All the OCD patients were un-medicated for at least 4 weeks. No patients were excluded due to their psychiatric comorbidity (including tic disorder) and they could participate if they had a primary diagnosis of OCD without predominant hoarding. Siblings were included provided that they did not meet lifetime criteria for OCD and had no current DSM-IV-TR axis I diagnosis. Healthy control subjects had no current DSM-IV-TR axis I diagnosis and no family history of OCD. The local ethical review board of VU university medical center approved all procedures and all subjects provided written informed consent.

### MRI acquisition

MRI was performed using a whole-body 3-Tesla MR system (Signa HDxt, GE Healthcare, Milwaukee, USA) equipped with an eight-channel phased-array head coil. Diffusion weighted echo-planar imaging was collected in 30 diffusion weighted (*b* = 1000 s/mm^2^) and five reference (*b* = 0 s/mm^2^) volumes with 49 contiguous axial slices of 2.4 mm slice thickness for covering the whole brain (repetition time TR 14 s, echo time TE 85 ms). The acquired in-plane resolution was 2.0 × 2.0 mm, which was reconstructed to 1.0 × 1.0 mm. Parallel imaging was applied with an acceleration factor of 2.

### Data processing

The diffusion MRI data were preprocessed for motion and eddy-current correction by using the FMRIB Software Library (FSL5; http://fsl.fmrib.ox.ac.uk/fsl). By fitting a tensor model to the raw diffusion data voxel-wise values of FA, AD, RD, and MD were obtained. All subjects' FA data were aligned to MNI standard space by using the non-linear image registration tool (FNIRT). Next, the mean FA image was created and thinned to create a mean FA skeleton, which represents the centers of all tracts common to the group. Each subject's aligned FA data was then projected onto this skeleton and the resulting data were fed into voxel-wise cross-subject statistics. The same non-linear registration was applied to the diffusivity parameters AD, RD and MD.

### Statistical analysis

We compared the skeletonized diffusion parameters between OCD patients, the unaffected siblings and healthy controls using a ROI approach, including the 7 ROI's (i.e., the corpus callosum, the bilateral cingulum bundle, the bilateral ILF/FOF, and the bilateral SLF) based on the results from the meta-analysis of Radua et al. (2014). The Johns Hopkins University (JHU) ICBM-DTI-81 white-matter labels atlas provided by FSL was chosen to define the ROIs. Permutation-based testing (5000 permutations) was carried out with Randomise, using Threshold-Free Cluster Enhancement (Smith and Nichols, 2009), and *p*-values were corrected for family wise error rate (FWE) taking into account multiple spatial comparisons. In addition to the ROI approach, for exploratory reasons, we performed whole-brain skeletonized voxel-wise statistics in order to check for group differences in the brain areas outside the ROIs. Corrected *p* < 0.05 was considered significant, and uncorrected *p* < 0.05 was considered a trend. Age and gender were added as covariates.

Randomise was used to compare FA values between OCD and HC. For each skeletonised ROI that had significantly different FA values between the two groups, we performed a three-group comparison, now including the unaffected siblings, using Randomise. Of voxels that showed difference in the three-group comparison, the mean diffusion parameters (for FA, AD, RD, and MD) were extracted for each subject (at uncorrected *p* < 0.05). Subsequently, One-way ANOVA analysis was performed to investigate the overall group effect (three groups) of AD, RD, and MD (*p* < 0.05). *Post-hoc* 2-sample-T tests were conducted to explore differences within groups. Within-group (OCD patients only) multiple regression analyses were carried out to investigate the relationship between FA and patients' disease severity (i.e., YBOCS severity scores).

## Results

### Demographic and clinical characteristics

Age, sex, education level and handedness did not differ significantly between OCD patients, unaffected siblings, and HC (see Table 1). A main effect of group was found for Y-BOCS (symptom list and severity scale), the OCI-R and MADRS. OCD patients scored higher compared with HC and the unaffected siblings (*p* < 0.01); the sibling group did not significantly differ from the HC group on these measures. Twenty-five OCD patients (57%) also met criteria for one or more comorbid current axis I diagnosis. No significant difference was found between OCD patients with (*n* = 25) or without (*n* = 19) comorbid diagnoses on demographic or other clinical measures (*p* > 0.16) (see Table 1).

**Table 1:** **Demographic and clinical characteristics of obsessive compulsive disorder patients, unaffected siblings, and healthy controls**.

	**OCD Patients (N = 44)**		**Siblings (N = 15)**		**HC (N = 37)**		**Analysis**	
	***N***	**%**	***N***	**%**	***N***	**%**	**X^2^ (df = 2) *p***	
**DEMOGRAPHIC MEASURES**								
Gender (men)	22	50	11	73	18	49		0.231
Handedness (right)	37	84	12	80	32	86		0.841
	**Mean**	**SD**	**Mean**	**SD**	**Mean**	**SD**	**F (df** = **2, 93)** ***p***	
Age (years)	38.5	9.9	38.1	14.1	39.5	11.5	0.1	0.893
Education level^a^	5.9	1.9	6.0	1.5	5.8	1.9	0.1^b^	0.953
**CLINICAL MEASURES**								
Y-BOCS severity (range 0–40)	21.48	6.16	0.33	0.03	0	0	84.96^b^	<0.001
OCI-R								
Total score	23.75	12.27	3.27	3.35	3.22	4.80	59.23^b^	<0.001
Washing score	2.77	3.84	0.20	0.41	0.30	0.62	14.17^b^	<0.001
Checking score	6.25	3.85	0.47	0.74	0.49	0.93	52.66^b^	<0.001
Order score	4.75	3.85	0.87	1.41	0.81	1.53	31.07^b^	<0.001
Obsession score	5.36	3.83	0.53	0.92	0.32	1.36	50.20^b^	<0.001
MADRS	11.66	8.54	1.93	3.77	0.89	1.51	52.60^b^	<0.001

a *Education level was recorded in nine levels ranging from 1 (no finished education) to 9 (university training)*.

b *Kruskal-Wallis test, H (df = 2, 93).Y-BOCS, Yale-Brown Obsessive Compulsive Scale; OCI-R, Obsessive-Compulsive Inventory-Revised; MADRS, Montgomery-Åsberg Depression Rating Scale; HC, Healthy Controls*.

### TBSS analysis: FA differences between OCD and HC

**Figure 1:**
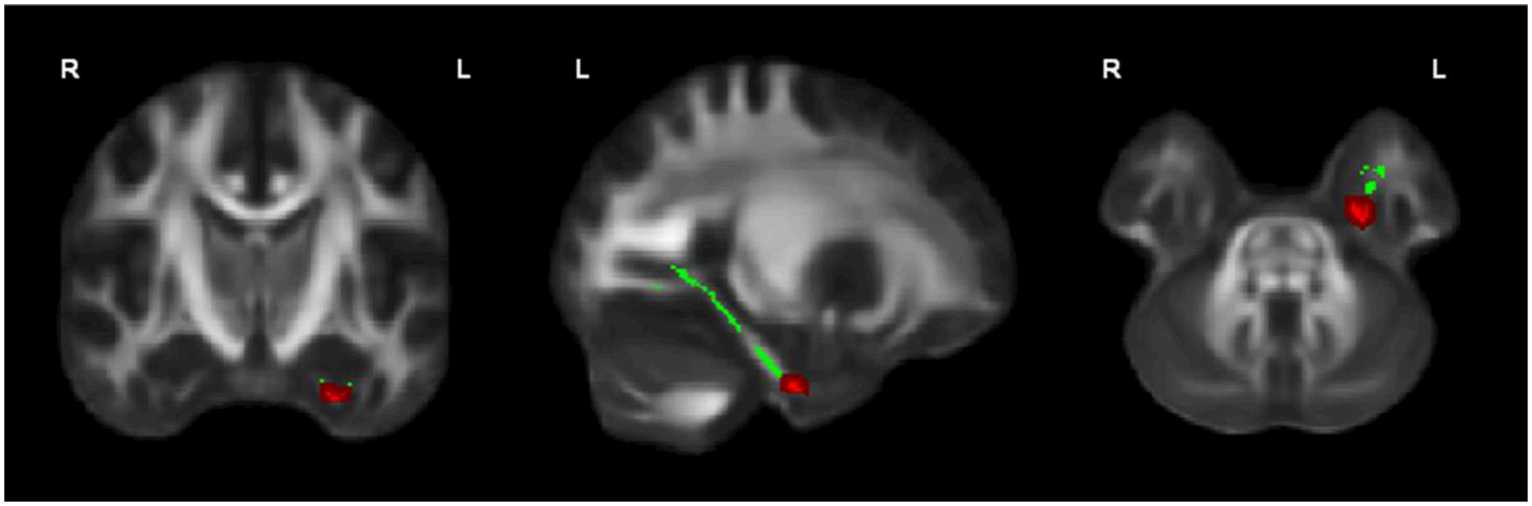
**OCD patients (***n*** = 44) showed significantly lower FA than healthy controls (***n*** = 37) in the left cingulum bundle, significant at ***p*** < 0.05 (in red; corrected for multiple comparisons); for illustration purposes, the displayed skeletonized results were thickened**. In green the parts of the skeleton within the pre-defined ROI.

**Figure 2:**
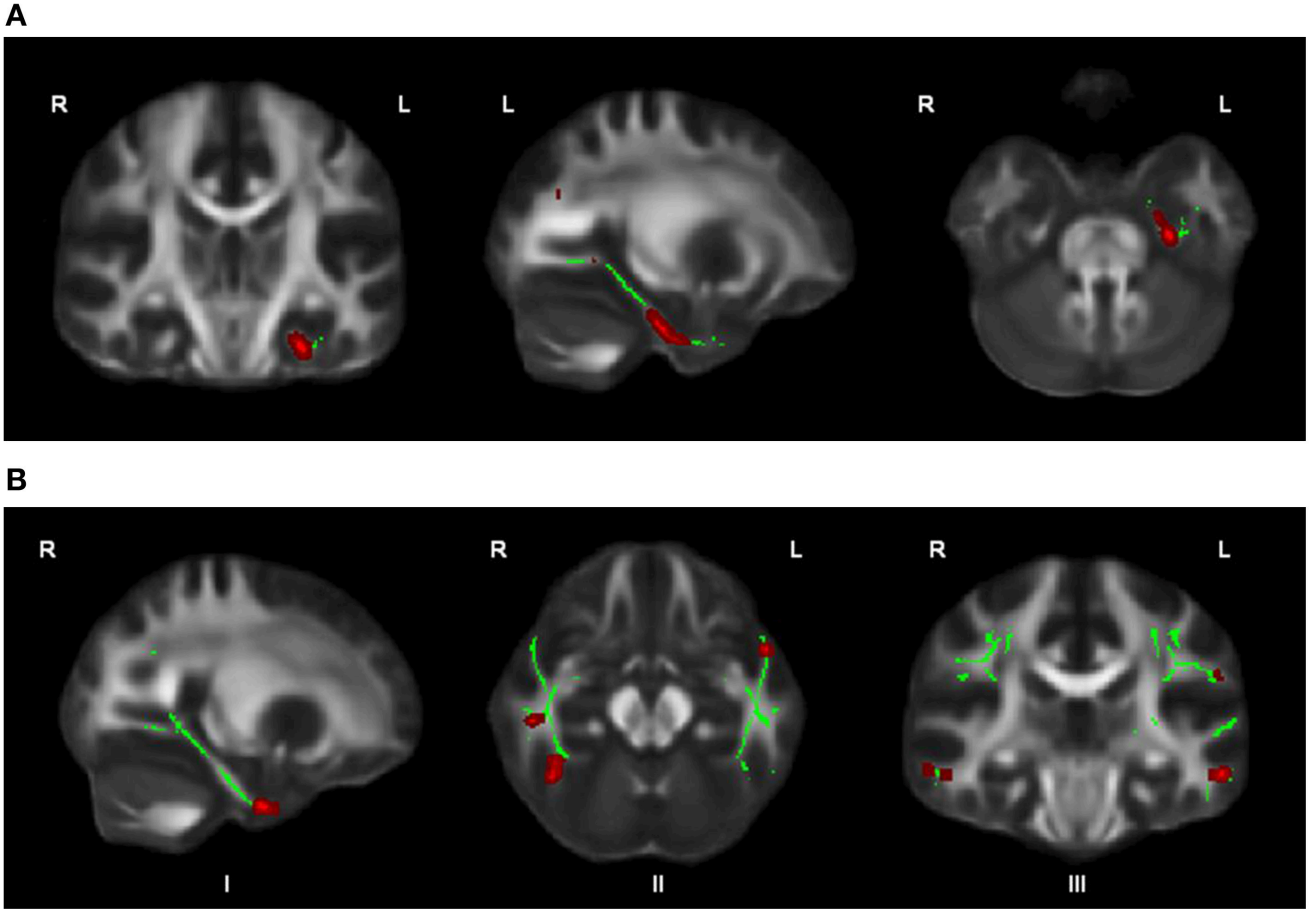
**OCD patients (***n*** = 44) compared with healthy controls (***n*** = 37) showed trend-significantly decreased FA in a larger part of the left cingulum bundle (A), in parts of the right cingulum bundle (I), bilateral ILF (II), and bilateral SLF (III) (in red; at ***p*** ≤ 0.05 uncorrected) (B); for illustration purposes, the displayed skeletonized results were thickened**. In green the parts of the skeleton within the pre-defined ROIs.

OCD patients, compared with HC, had significantly lower FA in the left cingulum bundle (peak MNI coordinates *x* = −24, *y* = −13, *z* = −32; ROI-corrected *p* < 0.05) as shown in Figure 1 and Table 2. At trend-significance level (uncorrected *p* < 0.05) OCD patients showed a lower FA in a larger area of the left and in parts of the right cingulum bundle, bilateral ILF, and bilateral SLF (see Figures 2A,B and Table 2). No difference in FA was found in corpus callosum (uncorrected *p* > 0.14). There were no areas in which OCD patients had higher FA than HC. TBSS analysis of the whole brain skeleton revealed no significant differences in FA between OCD and HC at the corrected level.

**Table 2:** **Locations of decreased FA in OCD patients compared to healthy controls**.

**ROIs**	**Peak coordinates**			**Cluster/Area**	**OCD < HC *p*-value (uncorrected)**
	***x***	***Y***	***z***		
Left ^*^ cingulum bundle	−24	−13	−32	38	0.001^*^
Right cingulum bundle	25	−5	−33	28	0.003
Left ILF/FOF	−37	−61	2	61	0.011
Right ILF/FOF	43	−50	−17	110	0.003
Left SLF	−51	−20	30	131	0.05
Right SLF	56	−12	−22	105	0.03

*Peak MNI coordinates, cluster sizes and p-values (uncorrected)*.

* *indicates significance at p < 0.05 corrected for multiple comparisons for ROI. FA, fractional anisotropy; IFO/FOF, inferior longitudinal fasciculus/frontal occipital fasciculus; SLF, superior longitudinal fasciculus; HC, healthy controls*.

YBOCS and OCI-R scores were found neither positively nor negatively correlated with FA within the OCD patients group.

### Three group comparisons—endophenotype analysis

**Figure 3:**
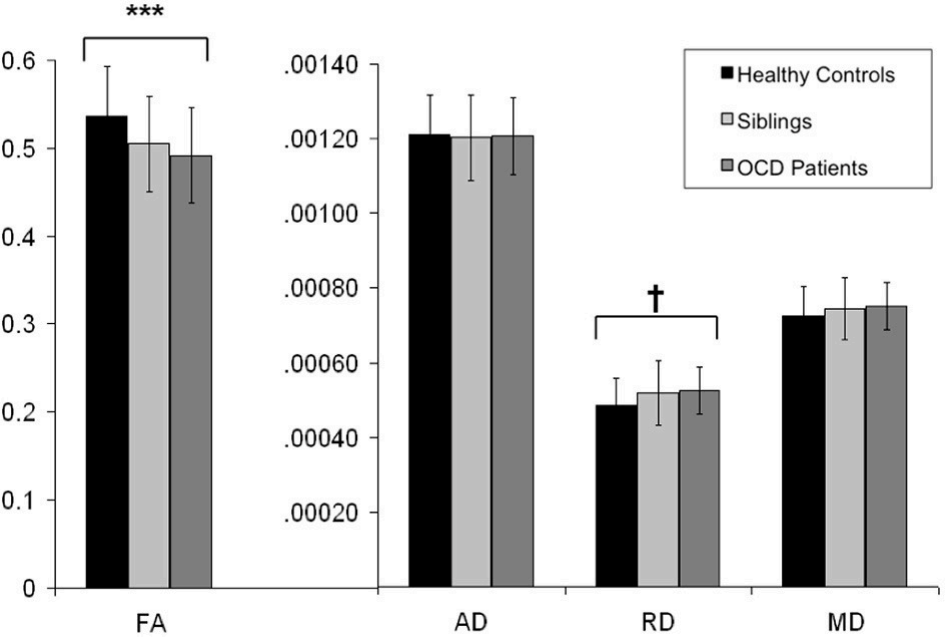
**Mean FA, AD, RD, and MD values from the extracted voxels in the left cingulum bundle across groups**. Y-axis indicates the mean FA, AD, RD, and MD values; along the X-axis are the three subject groups (healthy controls, unaffected siblings, and OCD patients). Solid dark bard represent healthy controls. Striped bars represent unaffected siblings. Light bars represent OCD patients. ^***^ indicates *p* < 0.001. ^†^ indicates *p* < 0.05 (trend significant). Error Bars: ± 1 SD. FA, fractional anisotropy; AD, axial diffusivity; RD, radial diffusivity; MD, mean diffusivity; OCD, obsessive-compulsive disorder.

FA of the left cingulum bundle showed a significant effect of group (peak MNI coordinates *x* = −28, *y* = −13, *z* = −31; ROI-corrected *p* < 0.05) as shown in Figure 1. Mean diffusion parameters values from the left cingulum bundle were extracted for all subjects based on voxels identified at uncorrected *p* < 0.05 in the [FA (three-group comparison)] F-contrast. We selected values within this larger area of the left cingulum bundle (167 voxels), since the significant area at corrected *p* < 0.05 is a relatively small cluster of 38 voxels located close to the inferior border of the cingulum bundle (see Figure 1). Figure 3 and Table 3 show that, after correction for multiple comparisons, mean FA was significantly lower in OCD patients compared with HC [*F*_(2, 93)_ = 6.40, *p* < 0.001]; mean FA values of unaffected siblings were intermediate between those of the OCD patients and of the HC, although *post-hoc* 2-sample-T comparisons did not show a significant difference between siblings and either the OCD patients or the HC (*p* = 0.223 and *p* = 1.000 respectively).

**Table 3:** **Means and standard deviations of FA, AD, RD, and MD values for subject groups of healthy controls, unaffected siblings and OCD patients in the left cingulum bundle**.

**Left cingulum bundle**	**HC *N* = 37 Mean ± SD**	**Siblings *N* = 15 Mean ± SD**	**OCD patients *N* = 44 Mean ± SD**
FA	0.54±0.06	0.51±0.05	0.49±0.05^***^
AD	120.9±10.9	120.2±11.4	120.8±10.3
RD	48.5±7.3	51.8±8.6	52.5±6.4
MD	72.6±7.7	74.6±8.3	75.2±6.3

*Diffusivities AD, MD, and RD are given in units of 10^−5^ mm^2^/s*.

*** *indicates p < .001 compared with healthy controls. AD, axial diffusivity; RD, radial diffusivity; MD, mean diffusivity; OCD, obsessive-compulsive disorder; HC, healthy controls*.

Mean values of AD and MD in the left cingulum bundle (see Table 3) did not significantly differ across groups. RD values in this region showed a reverse pattern compared to FA values, with the OCD patients showing trend-significantly higher RD compared with HC and the unaffected siblings representing an intermediate group (see Table 3 and Figure 3). Although no effect of group was found for these diffusivity values after correction for multiple comparisons, a trend-significant effect of group was observed for the RD values [*F*_(2, 93)_ = 3.34, *p* = 0.04 uncorrected]. To test laterality of these results, we performed the same procedure for the diffusivity values in the right cingulum bundle, showing a similar pattern, although it does not surpass the trend-significant level.

## Discussion

The main finding of the present study is lower FA in the left cingulum bundle in un-medicated OCD patients compared with HC, which partially replicates the findings from the meta-analysis conducted by Radua et al. (2014). We also found, in line with Radua et al., although at uncorrected significance level, lower FA in the other ROIs (except for the corpus callosum), i.e., the right cingulum bundle, the bilateral ILF/FOF, and the bilateral SLF. Lower FA appeared to be associated with higher RD. The three-group comparison showed that the unaffected siblings seemed to represent an intermediate group between the OCD patients and HC with respect to FA in the left cingulum bundle.

Lower FA in the cingulum bundle has been reported by most studies that investigated adult OCD patients (Koch et al., 2014; Radua et al., 2014). Increasing evidence from structural and functional neuroimaging research in recent years has emphasized the impact of deficits in temporo-parietal-occipital regions in the pathophysiology of OCD besides the frontal-striatal and fronto-limbic neurocircuitries (Menzies et al., 2008a; Piras et al., 2013). The cingulum bundle contains many short and long association fibers linking the frontal lobe with the temporal lobe (Schmahmann and Pandya, 2006), supporting communication between the prefrontal, parietal, and temporal regions (Jones et al., 2012). The finding of lower FA in the left cingulum bundle in OCD supports the suggested involvement of temporal and parietal regions in the pathophysiology of the disorder.

Although FA values in the right cingulum bundle, ILF/IFOF and SLF were only trend-wise lower in OCD patients compared with HC, these findings may still be relevant since these differences were observed in all a-priori hypothesized regions bilaterally.

In general, the changes in diffusion parameters observed in these un-medicated OCD patients were subtle. Although Radua et al. (2014) in their meta-analyses reported widespread white matter abnormalities in OCD, no clear evidence so far has suggested OCD as a typical white matter disease (Koch et al., 2014). Furthermore, the lack of consistent DTI findings in the OCD literature may be explained, at least partly, by the impact of medication in OCD. Radua et al. (2014) reported that the lower FA was most prominent in samples with more medicated OCD patients. Benedetti et al. (2013) found that medicated OCD patients had higher RD in the corpus callosum and adjacent cingulate gyrus when compared to drug-naïve OCD patients and HC. Other studies in OCD also showed pharmacological treatment effects on white matter alterations (Yoo et al., 2007; Fan et al., 2012). Therefore, the subtle white matter abnormalities found in our un-medicated patient sample suggests that medication might be a potential confounder in most previous studies.

The here reported (trend-) significantly lower FA values all concern white matter regions that are spatially connected to temporal-parietal-occipital regions. Structural abnormalities found in both gray matter and white matter regions in posterior parts of the brain of OCD patients mainly concern the parietal lobe extending to the temporal and occipital lobes (Piras et al., 2013). These posterior regions are associated with cognitive functions including visuo-spatial functions, which have been consistently found to be impaired in OCD patients (Cohen et al., 1996; Savage et al., 1999). Our findings thus indirectly suggest abnormalities in these white matter microstructures might also be contributors to the cognitive impairments in visuo-spatial abilities.

The three-group comparison on FA in the left cingulum bundle showed a significant effect of group, with the unaffected siblings showing intermediate FA values. Mean AD and MD values did not differ across groups. Mean RD revealed a reverse pattern to that seen for FA: RD was trend-significantly higher in OCD patients compared to HC, with intermediate values in unaffected siblings. Such changes in diffusion parameters are in line with findings from previous DTI studies in OCD (Bora et al., 2011; Fan et al., 2012). Although Song et al. (2003) suggested a higher RD to be associated with demyelination, others have shown that the interpretation of changes in RD and AD needs careful consideration, due to the inherent disbalance between fiber thickness and imaging resolution (Wheeler-Kingshott and Cercignani, 2009). Although a few genetic studies suggested myelination in the OCD pathophysiology (Zai et al., 2004; Stewart et al., 2007), we can only speculate about possible disruption of myelin integrity contributing to the white matter abnormalities in the left cingulum bundle. A similar pattern of lower FA in combination with higher RD (and normal AD values) has also been reported for autism, schizophrenia, and depression (Alexander et al., 2007; Ashtari et al., 2007; Lee et al., 2007; Michael et al., 2008; Seal et al., 2008; Whitford et al., 2010; Korgaonkar et al., 2011).

With regard to both FA and RD, the unaffected siblings seem to represent an intermediate group between patients and HC, suggesting that white matter alterations can be considered, at least partly, an endophenotype of OCD. The identification of disease endophenotypes can help to identify possible genetic risk factors of diseases and environmental effects. Menzies et al. (2008b) also found abnormal FA values in OCD patients as well as their first-degree relatives, although in that study abnormalities were mainly reported in the parietal and medial frontal regions.

To our knowledge, this is the first study investigating the white matter endophenotype of un-medicated OCD by exploring FA in combination with AD and RD. The sample sizes of the OCD and control groups were fairly large compared to previous studies and the ROIs were selected based on the most recent meta-analysis (Radua et al., 2014). An important limitation of the present study is that the reported findings were not corrected for multiple comparisons as we identified 7 ROIs *a priori*. In addition, we can't exclude the long-term effects of past medication use, although a 4-week washout period is thought as a sufficient time period for wearing off direct medication effects. Finally, only 15 unaffected siblings were included in the study. Due to the limited sample size, we can't rule out the possibility that the observation that the unaffected siblings were neither significantly different from OCD patients nor the HC might be due to the limited statistical power.

In conclusion, this DTI study shows white matter alterations in OCD patients, un-medicated for at least 4 weeks, compared with HC, mainly in the left cingulum bundle. A lower FA seems to be related to trend-wise higher RD, suggesting potential disruption of myelin integrity in this region. The fact that the unaffected siblings represent an intermediate group between OCD patients and HC is suggestive for a white matter endophenotype of OCD, reflecting genetic vulnerability.

## Author contributions

SF contributed to data analysis and paper writing. OH contributed to daily supervision on the research analyses and paper writing. DC, YV, and DV contributed to supervision on the research. SD and FD contributed to data collection. PP contributed to technical supports and daily supervision on the research analyses and paper writing.

### Conflict of interest statement

The authors declare that the research was conducted in the absence of any commercial or financial relationships that could be construed as a potential conflict of interest. The Associate Editor Kirsten R Müller-Vahl declares that, despite of having collaborated with Danielle C. Cath in the EU funded MC-ITN TS-Eurotrain, the review process was handled objectively.
